# Long-Term Functional Outcomes Following Enzymatic Debridement of Deep Hand Burns Using Nexobrid^®^: A Retrospective Analysis

**DOI:** 10.3390/jcm13164729

**Published:** 2024-08-12

**Authors:** Asja T. Malsagova, Amin El-Habbassi, Moritz Billner, Maresa Berns, Tamas Pueski, Karl J. Bodenschatz, Paul I. Heidekrueger, Denis Ehrl

**Affiliations:** 1 Department for Plastic, Reconstructive and Hand Surgery, Burn Center for Severe Burn Injuries, Nuremberg Hospital, Paracelsus Medical University, Breslauer Str. 201, 90471 Nuremberg/Prof.-Ernst-Nathan Straße 1, 90419 Nuremberg, Germany; moritz.billner@klinikum-nuernberg.de (M.B.); maresa.berns@klinikum-nuernberg.de (M.B.); tamas.pueski@klinikum-nuernberg.de (T.P.); denis.ehrl@klinikum-nuernberg.de (D.E.); 2 Paracelsus Medical University Salzburg, Muellner Hauptstr. 48, 5020 Salzburg, Austria; amin.habbassi@stud.pmu.ac.at; 3 Department for Pediatric Surgery, Nuremberg Hospital, Paracelsus Medical University, Breslauer Str. 201, 90471 Nuremberg/Prof.-Ernst-Nathan Straße 1, 90419 Nuremberg, Germany; karl.bodenschatz@klinikum-nuernberg.de; 4 Centre of Plastic, Aesthetic, Hand and Reconstructive Surgery, University of Regensburg, Universitätsstraße 31, 93053 Regensburg, Germany; paul@heidekrueger.net

**Keywords:** enzymatic debridement, Nexobrid^®^, hand burns, hand function

## Abstract

**Background**: For years, surgical debridement with autografting has been considered the standard of care in the treatment of severe burns of the hand. However, in recent years, enzymatic debridement has increasingly been reported as a good alternative, especially for burns of the hand, as it selectively preserves viable tissue. In this study, we aim to evaluate the long-term function of the hand after enzymatic debridement in deep dermal burns. **Methods**: A retrospective chart review was conducted as well as measurements of subjective and objective outcome measures through physical examination and Disabilities of the Arm, Shoulder, and Hand (DASH), Patient and Observer Scar Assessment Scale (POSAS), and Vancouver Scar Scale (VSS) scores. **Results**: A total of 32 enzymatically debrided hands of 24 patients were included with a mean age of 42.4 ± 16.8 years and a mean follow-up of 31 months. Postoperatively, 19 of these could be managed conservatively using skin substitutes such as “Suprathel”, 13 had to undergo subsequent autografting. The mean DASH score for the entire study population was eight with a mean value of four in the conservatively managed group and fourteen in the autografted group. The mean Patient, Observer POSAS, and VSS values were nineteen, thirteen, and two. A total of 30 cases showed an effortless complete fist closure, and, also in 30 cases, patients attested to be satisfied with the esthetic appearance of the hand on being asked. **Conclusions**: The descriptive analysis of these results in our study population suggests that the enzymatic debridement of deep burns of the hand, especially combined with subsequent conservative management with skin substitutes, was associated with low long-term hand disability scores at a follow-up of two years.

## 1. Introduction

Over the past decade, bromelain-based enzymatic debridement has established itself as an effective and even superior alternative to conventional excisional debridement, preventing the necessity of escharotomy, reducing the number of burns requiring autografting, as well as reducing healing time from the first debridement [1,2,3,4,5,6,7]. In many clinics, it is steadily replacing conventional debridement as the standard of care in the treatment of deep dermal burns because of its selective ability to preserve (partially) viable dermal tissue and enhance the chances of spontaneous healing [4,8,9].

Hand burns are very common, being implicated in 30 to 90% of burn patients [2,10,11,12], and are an especially challenging field within burn treatment because of the hand’s complex anatomy. Especially in this field, enzymatic debridement has been of great significance. Preserving viable dermal tissue in the hand, a complex anatomical instrument prone to developing contractures due to its many joints can be beneficial for maintaining normal hand function.

With an increasing number of burn victims surviving their injuries, the preservation of hand function becomes even more crucial for their quality of life [13]. Conventional excisional debridement in the form of tangential excision or hydrosurgery is very traumatic and inadvertently removes healthy dermal tissue with its capacity for spontaneous healing. The often-used aphorism “If epidermis is life, dermis is quality of life” is particularly pertinent in this field of burn surgery, for having one normally or nearly normally functioning hand can make the difference between being self-sufficient and being dependent on others in our daily activities.

The early and continuous intensive mobilization of the hand after deep burn injuries is essential for long-term hand function [10,12,14,15]. Enzymatic debridement seems to not only preserve viable dermal tissue optimizing the patient’s own regeneration potential, but also spares the patient the postoperative hand immobilization. Studies evaluating long-term hand functionality after enzymatic debridement are scarce. These find similar or superior long-term functional results after enzymatic debridement in comparison to conventional debridement in adults [2,4,7]. In our study, we aimed to evaluate the long-term hand functionality in our adult patient population after the enzymatic debridement of deep dermal burns.

## 2. Materials and Methods

This study has been approved by our hospital’s Institutional Review Board. A retrospective analysis was conducted on patients having undergone enzymatic debridement of the hands between 2015 and 2022 in our inpatient clinic (with 26 beds) or the intensive care unit for severe burns (with 6 beds) in “Nuremberg Hospital”. “Klinikum Nuremberg” is a 2200-bed tertiary hospital.

### 2.1. Patient Inclusion

**Figure 1:**
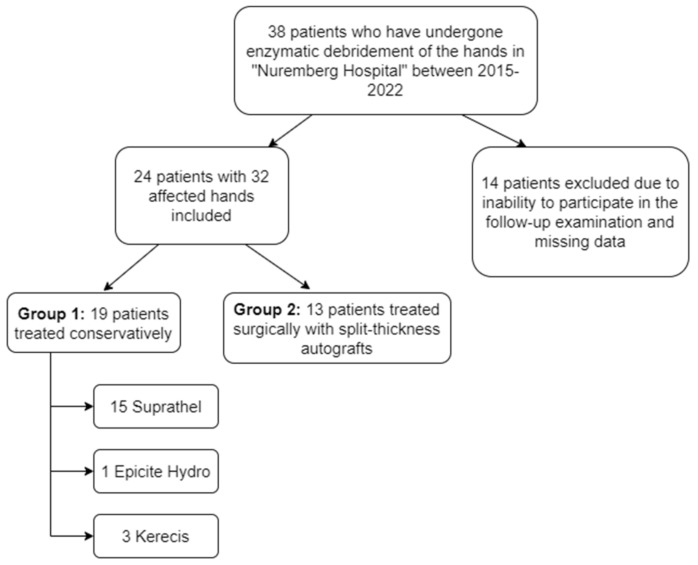
Flowchart of patients.

All cases of deep hand burns in which enzymatic debridement with Nexobrid^®^ was applied were searched and identified in the clinics database using specific International Classification of Disease (ICD) codes. Our study population and this study only included patients over the age of 16. Patients with burns under the age of 16 are treated by pediatric surgeons in our clinic. We aimed to exclude individuals with rheumatoid diseases impacting the hands, as well as those with other hand traumas or a history of hand surgery that affected their hand function, as these conditions would introduce a potential bias. However, none had to be excluded for these reasons. A total of 38 patients had been selected and 24 of them were included in the study. For the remaining 14 patients, the distance from their home to our clinic was too far, they were unable to fit the visit into their schedule during the weeks when we were available to perform physical examinations, or the retrospective chart review showed too many significant missing variables (see the flowchart in Figure 1).

### 2.2. Standard Procedures in Regards to Enzymatic Debridement

On admission, the included patients with deep hand burns received wet wound dressings consisting of bandages rinsed in polyhexanide solution. These wet dressings were continued for at least 24 h as part of the presoaking regime. In our clinic, burns are diagnosed and designated using capillary refill testing as well as Laser Doppler Imaging (LDI).

Enzymatic debridement with Nexobrid^®^ in our clinic follows European consensus guidelines [16,17]. Depending on the initial wound evaluation post-debridement (24–48 h), the following treatment paths were determined: superficial burns were treated conservatively through “Suprathel” application, while deep partial-thickness and mixed burns were treated conservatively with skin substitutes “Epicite Hydro” or “Kerecis”. Full-thickness burns required split-thickness autologous skin grafting. Mixed-depth burns were also managed conservatively to promote spontaneous epithelialization (see flowchart in Figure 1). If healing was inadequate after 2 weeks, autografting was considered. Early mobilization was encouraged for the conservatively treated cases (starting on the first postoperative day), while the autografted hands were immobilized for 4–5 days postoperatively.

### 2.3. Data Collection and Final Routine Follow-Up Examination

A retrospective data analysis was performed including reviewing the patient files and photo and video documentation. In the period between March 2022 and August 2022, the included patients were invited to participate in a routine follow-up examination. During the routine follow-up examination, the patients were asked to fill out the Disabilities of the Arm, Shoulder, and Hand (DASH) [18,19], Patient and Observer Scar Assessment Scale (POSAS) [20,21], and Vancouver Scar Scale (VSS) [22] scores. Additionally, patients were interviewed regarding current symptoms, persisting disabilities during daily life, their professional situation, their medical history, and whether they were satisfied with the esthetic appearance of their affected hands or not. This examination included a detailed physical assessment of various aspects of hand function. The condition of the scars and any potential contractures were carefully evaluated. Sensitivity was assessed using the two-point discrimination test, a method that determines the ability to discern two close points on the skin. To evaluate thumb mobility, the opposition score as defined by Kapandji [23] was employed, which provides a standardized measure of thumb function. The range of motion (ROM) of both the metacarpophalangeal (MCP) and interphalangeal (IP) joints of the affected fingers was measured using a goniometer. Additionally, hand strength was assessed using specific tools. Pinch strength between the thumb and index finger was measured with the Jamar pinch gauge, which is designed to evaluate the force exerted in a person’s pinch grip. Overall, handgrip strength was measured using the Jamar dynamometer, an instrument for assessing the maximum isometric strength of the hand and forearm muscles. Most importantly, patients were instructed to demonstrate full fist closure and full hand extension, both of which were documented photographically. The physical examinations were conducted by a fifth-year medical student under the direct supervision of the first author, following extensive practice of the procedures.

### 2.4. Statistical Analysis

The relatively low number of patients in the conservatively and surgically treated groups after enzymatic debridement caused a proper statistical analysis to be impossible. Therefore, only a descriptive analysis was performed.

## 3. Results

A total of 24 patients were included in this study, with 32 enzymatically debrided burned hands and with a mean follow-up of 31 months. The mean age of these patients was 42 years (ranging from 16 to 71), with twenty-one male and three female patients (Table 1). In eight patients, both hands were involved, and in nineteen patients, the dominant hand was involved. The mean total burned body surface area (TBSA) was 15%, ranging from 10 to 70%. A total of six hands had mixed burns with superficial partial-thickness being predominant; eighteen showed deep partial-thickness burns; and eight showed full-thickness burns. In the case of a mixed burn pattern, the predominant burn depth was documented. In 22 hand burns in our study, both the palm and the dorsum of the hand were involved.

After enzymatic debridement, 19 hands were treated conservatively (group 1) and 13 hands surgically (group 2). Both groups did not show relevant differences in mean age or follow-up time and consisted of predominantly men. The mean TBSA was greater in group 2 with 23% vs. 10% in group 1. Also, group 2 included seven out of the total eight full-thickness burns. The palm of the hand seemed to be more frequently affected in group 2 (12/13 vs. 14/19).

Some observations in the Results Section warrant further clarification. Despite the study’s primary focus on deep dermal burns, Table 1 indicates the inclusion of six superficial partial-thickness burns. This discrepancy arises from the classification of many mixed-depth burn cases by their predominant depth. Specifically, these six cases consisted of a mix of 60% superficial partial-thickness and 40% deep partial-thickness and/or full-thickness burns. None of these six mixed-depth burn cases had to be treated surgically.

### 3.1. Conservative vs. Surgical Treatment Following Enzymatic Debridement

A total of nineteen hand burns were treated conservatively, fifteen of which with “Suprathel”, three of which with “Kerecis”, and one with “Epicite Hydro” (Table 2). The 13 hand burns in group 2 were all treated surgically following enzymatic debridement through excisional debridement and split-thickness autografting. In twelve cases, the indication for ensuing surgical treatment was set directly at the post-enzymatic wound bed evaluation, and, in one case, after the failed conservative treatment.

In 10 out of 12 cases where a pink or red wound bed evaluation (superficial partial-thickness burns) was observed, a conservative treatment also ensued. The two remaining cases, despite initially presenting with a uniform pink wound bed upon evaluation, exhibited delayed healing in the following 2 weeks and consequently required subsequent surgical treatment. Also, in all but one case where signs of full-thickness burns were identified (such as step-offs/depressions, exposed fatty tissue, and thrombosed veins), surgical treatment with split-thickness autografting ensued. A white wound bed with pin-point punctate bleeding (deep partial-thickness burns), occurring in eighteen cases, resulted in conservative treatment in seven of the cases and surgical treatment in eleven cases.

### 3.2. Questionnaire Scores

**Figure 2:**
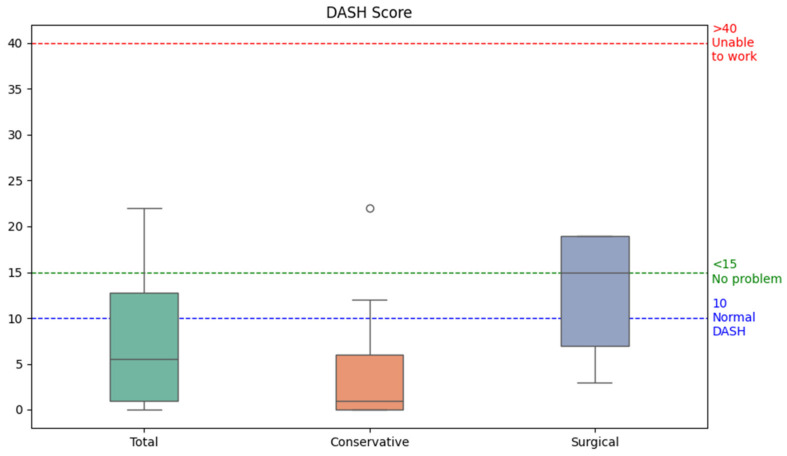
Boxplots for the Disabilities of the Arm, Shoulder, and Hand (DASH) score distribution of the conservatively and surgically treated groups following enzymatic debridement. The dot in the middle of the figure is an outlier value. The normal DASH score has been reported to be around 10 in a general population study [24]. The following cut-off values for the DASH score have been described: <15 corresponds with “no problem”, 16–40 with “problem, but working”, >40 with “unable to work” [25].

The mean DASH score was found to be eight for the entire study population (range 0–27), with a mean score of four in the conservatively treated group and a mean score of fourteen in the surgically treated group (Table 3, Figure 2). A total of nine patients (28%) had a DASH score ≥ 15, eight of which were treated surgically and one conservatively. The DASH score seemed not to be age-dependent.

**Figure 3:**
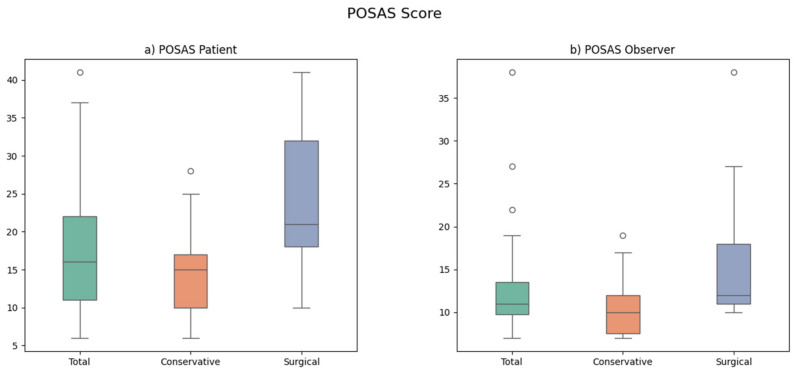
Boxplots for the Patient (**a**) and Observer (**b**) Scar Assessment Scale (POSAS) of the conservatively and surgically treated groups following enzymatic debridement. The dots represent outlier values.

The mean patient POSAS score was 19 for the entire study population, with a mean value of 14 in the conservatively treated group and 24 in the surgically treated group. The mean values for the observer POSAS score were 13 for the entire study population, 10 in group 1, and 17 in group 2 (Table 3, Figure 3). The mean VSS score was two in the entire group, with a mean value of two in the conservatively treated patients and four in the surgically treated patients. A total of two patients attested not to be pleased with the esthetic appearance of their burned hand. Both of them were treated surgically following enzymatic debridement.

### 3.3. Functional Results

In four cases, pain was reported involving the burned hand area (one case in group 1 and three cases in group 2). Cold intolerance was reported in fourteen cases, eight of which were in the surgically treated group. In 31 cases, a two-point discrimination between 4 and 6 mm was found. Scar contractures were seen in a total of nine burned hands, seven of which were in the surgically treated group. These included mostly interdigital contractures.

**Figure 4:**
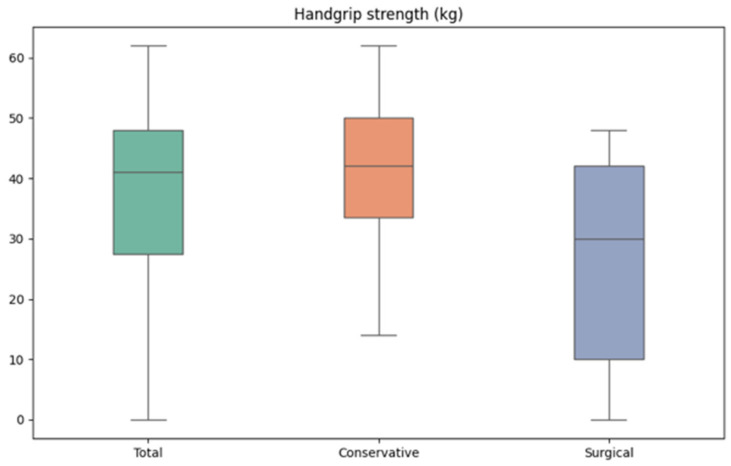
Boxplots for the handgrip strength of the conservatively and surgically treated groups following enzymatic debridement.

Nonetheless, in 30 of the 32 cases, the patient could demonstrate complete fist closure. Most hands showed an overall full range of motion, with nearly full extension and the abduction of the fingers. A maximum Kapandji score of ten was found in twenty-five hands and a score of nine was found in six hands. In one surgically treated hand, a Kapandji score of four was found. The mean handgrip strength was found to be 36 kg for the entire group, with 41 kg in group 1 and 28 kg in group 2 (Figure 4). The mean pinch grip strength was also lower in group 2 with 6 kg compared to 8 kg in group 1.

## 4. Discussion

In this study, we aimed to evaluate the long-term hand function after the enzymatic debridement of deep dermal burns through descriptively analyzing the following outcome measures and comparing them to the existing literature.

### 4.1. DASH

For the DASH questionnaire, we found a mean score of eight, (four in group 1, fourteen in group 2). Our mean DASH score of eight is beneath the score of ten, which was found in a general study population [24], and beneath the cut-off value of 15, which is considered unproblematic in the DASH score interpretation [25]. In a prospective study of 16 enzymatically debrided upper extremities, Cordts et al. found a mean DASH score of 23 at a 3-month follow-up [11]. This is relatively high in comparison to many other similar studies, which have a longer follow-up time, such as Fischer et al., who found a mean DASH of 13.9 in a prospective study of 20 enzymatically debrided upper extremities at a 12-month follow-up [3]. This role of the follow-up time is confirmed by Cherubino et al., finding a mean DASH of 21 at a 6-month follow-up and a mean DASH of 11 at a 15-month follow-up in 18 enzymatically debrided upper extremities [26]. Furthermore, Corrales-Benitez et al. prospectively found a mean DASH value of 0.2 in 90 enzymatically debrided hands [27]. Heitzmann et al. reported a mean DASH score of seven in thirty-one enzymatically debrided hands. Both of these were found at a mean follow-up of 12 months, which is very similar to our own results [4]. Despite the many inhomogeneities of these studies, they do seem to support the favorable DASH values found in our study, especially after a follow-up time of 12 months or longer.

### 4.2. POSAS and VSS

With a mean patient POSAS value of 19 and an observer POSAS value of 13, one could conclude that, in our study, the observers were generally more satisfied with the long-term scars than the patients were. However, the POSAS values suggest that both patient (POSAS of 14 in group 1 vs. 24 in group 2) and observer (POSAS of 10 in group 1 vs. 17 in group 2) seemed to be more satisfied with the scar quality after conservative post-enzymatic treatment than after surgical post-enzymatic treatment (Table 3). In the study by Cherubino et al., mean patient and observer POSAS values of 16 and 14 were reported [26]. Heitzmann et al. conducted a comparative study between 31 enzymatically and 15 surgically debrided hands, reporting patient and observer POSAS values of 21 and 17, respectively in the enzymatically debrided group, and of 33 and 26 in the surgically debrided group [4]. These patient and observer POSAS values suggest poorer scar quality after surgical debridement than after enzymatic debridement of hand burns and seem to correspond with our own results.

The mean VSS value of two (of thirteen) found in this study suggests an overall favorable subjective scar quality, with a worse score in the surgically treated group 2. The minimal difference in VSS scores between groups 1 and 2 (two vs. four), however, is not very interpretable. The aforementioned study by Cordts et al. finds a mean VSS score of six in enzymatically debrided hands [11], corresponding with a worse scar quality than in our study, however at a much shorter follow-up.

### 4.3. Fist Closure and Handgrip Strength

Only two of the thirty-two examined hands were unable to demonstrate a full fist closure at a mean follow-up of 31 months, suggesting a relatively good long-term hand function. Both of these hands were treated surgically post-enzymatically, arguing in favor of conservative post-enzymatic treatment. A general population study by Wang et al. in the USA reports a mean grip strength of 40 kg in the dominant hand of men [28]. Considering the fact that our study concerned injured hands of mostly men with 80% of the study population, a mean grip strength of 36 kg (41 kg in group 1 vs. 28 kg in group 2) seems acceptable. As of yet, the literature does not provide many studies reporting on the handgrip strength after enzymatic debridement.

In line with our findings, most of these previous studies [3,5,6,11,26,27,29], especially the ones comparing enzymatic debridement to the standard of care [1,2,4,7], find long-term functional and esthetic results which are equal or superior to conventional excisional debridement.

### 4.4. Conservative vs. Surgical Treatment Following Enzymatic Debridement

In this study, we were not able to compare enzymatically debrided hands to conventionally debrided hands; however, we were able to descriptively compare our subgroups consisting of the conservatively and surgically treated hand burns post-enzymatically. Most of the functional outcome measures in this study (Table 3) were worse in the surgically treated group following enzymatic debridement. In particular, the differences in DASH and POSAS scores, fist closure, and handgrip strength were noteworthy. The relatively high number of deep partial-thickness burns in the conservatively treated group suggests that, on initial presentation, seemingly deep burns can be managed conservatively, confirming the reduction in autografting after enzymatic debridement reported in previous studies [1,2,7].

However, to our knowledge, we seem to be the first to compare conservative and surgical treatment modalities following enzymatic debridement. We consistently found worse esthetic and functional results in the surgically treated group than in the conservatively treated group post-enzymatically. This corresponds with the trend seen in the comparison between primarily surgically debrided hand burns vs. primarily enzymatically debrided hand burns. This is illustrated, for instance, by the parallel between the better POSAS values in the conservatively treated group (14 and 10 in group 1 vs. 24 and 17 in group 2) in our study and the better POSAS values in the enzymatically debrided group (21 and 17 in the enzymatic group vs. 33 and 26 in the surgical group) in the study by Heitzmann et al. [4]. Therefore, a logical hypothesis seems to be that, when hand burns do require surgical treatment either indicated directly at presentation, after post-enzymatic wound bed evaluation, or after failed conservative treatment, the pretreatment through enzymatic debridement seems to become less important for long-term hand function for the surgical trauma of excisional debridement and the contracture potential of autografts remain the same. Therefore, the functional results of surgically treated hand burns post-enzymatically might be comparable to the functional results after the conventional surgical treatment of hand burns. Of course, this hypothesis warrants further research in a comparative study with conventionally surgically treated deep dermal hand burns.

### 4.5. Limitations

An important limitation of this study is its retrospective nature and the subsequent difficulties in obtaining homogenous data. This study has a relatively extended follow-up period of 31 months and focuses exclusively on enzymatically debrided hands.

However, for a statistical analysis, the sample size of both groups is too small to yield reliable statistical testing. Our conclusions are based on a descriptive analysis only, and should therefore be interpreted with caution. These trends need to be validated through future research involving larger sample sizes that would allow for meaningful statistical analysis. The absence of a control group consisting of primarily surgically debrided hand burns also poses a potential limitation, as mentioned above. Additionally, the greater mean TBSA and the number of full-thickness burns in group 2 pose a potential bias.

## 5. Conclusions

With a relatively low sample size, the enzymatic debridement of deep dermal hand burns in this study showed a promising trend in long-term functional results, especially if it is followed by conservative wound treatment using skin substitutes. With complete fist closure achieved in nearly all patients and mean DASH scores indicating low disability levels, we argue that enzymatic debridement is a viable treatment method for hand burns, where the preservation of dermal tissue can be crucial for the maintenance and restoration of hand function after deep dermal burns.

## Figures and Tables

**Table 1:** Patient characteristics presented with mean values and standard deviations. (Chronic obstructive pulmonary disease (COPD)).

	Total Study Population (*n* = 32)	Conservatively Treated Group 1 (*n* = 19)	Surgically Treated Group 2 (*n* = 13)
Age (years)	42.4 ± 16.8	43	41
Follow-up (months)	31.4 ± 17.1	32	31
Male sex	26	17	9
Total burned body surface area (%)	15.1 ± 15.3	10	23
Burn depth			
Superficial partial-thickness	6	6	0
Deep partial-thickness	18	12	6
Full-thickness	8	1	7
Palm of the hand involved	26	14	12
Dorsum of the hand involved	28	17	11
Renal insufficiency	4	3	1
Heart disease	0	0	0
Immunosuppression	0	0	0
COPD	3	3	0
Depression	1	1	0
Nicotine dependency	6	6	0

**Table 2:** Specifics pertaining to the enzymatic debridement and the ensuing conservative or surgical treatments.

	Total Study Population (*n* = 32)	Conservatively Treated Group 1 (*n* = 19)	Surgically Treated Group 2 (*n* = 13)
Wound bed:			
Uniform pink/red	12	10	2
Uniform white + punctuate bleeding	18	7	11
Step-off/Depression	6	0	6
Exposed fatty tissue	4	0	4
Exposed thrombosed veins	6	1	5
Second applicatiion of Nexobrid^®^	0	0	0
Post-enzymatic application of			
Suprathel	15	15	0
Epicite Hydro	1	1	0
Kerecis	3	3	0
Split-thickness autograft	13	0	13

**Table 3:** Questionnaire scores and functional outcomes. Disabilities of the Arm, Shoulder, and Hand (DASH), Patient and Observer Scar Assessment Scale (POSAS), Vancouver Scar Scale (VSS).

	Total Study Population (*n*= 32)	Conservatively Treated Group 1 (*n* = 19)	Surgically Treated Group 2 (*n* = 13)
DASH (0–100)	8.4 ± 8.1	4	14
POSAS Patient (6–60)	18.5 ± 9.1	14	24
POSAS Observer (6–60)	12.9 ± 6.5	10	17
VSS (0–13)	2.4 ± 1.7	2	4
Complete fist closure			
possible	30	19	11
not possible	2	0	2
Kapandji Score (0–10)	10	10	9
Handgrip strength (kg)	35.8 ± 16.2	41	28
Pinch grip strength (kg)	7.1 ± 3.1	8	6

## Data Availability

All the data analyzed during this study are available from the corresponding author on reasonable request.
